# Editorial: Role of lipids in virus assembly

**DOI:** 10.3389/fmicb.2015.00410

**Published:** 2015-05-05

**Authors:** Jamil S. Saad, Delphine M. Muriaux

**Affiliations:** ^1^Saad Lab, Microbiology, University of Alabama at BirminghamBirmingham, AL, USA; ^2^Centre D'études D'agents Pathogènes et Biotechnologies Pour la SantéMontpellier, France

**Keywords:** retroviruses, HIV-1, Gag, Matrix, membrane, NMR, Ebola, PI(4, 5)P2

Viruses utilize cellular lipids during critical steps of replication like entry, assembly, and egress. Growing evidence indicate important roles for lipids and lipid nanodomains in virus assembly. This special topic covers key aspects of virus-membrane interactions during assembly and egress of two classes, retroviruses and filoviruses. It discusses molecular mechanisms of assembly and budding of retroviruses and Ebola virus (EBOV) and how various membrane components facilitate these events. It is well established that assembly of most of retroviral Gag proteins occurs on the plasma membrane (PM) (Ono et al., 2004; Grigorov et al., 2006; Jouvenet et al., 2006, 2008; Finzi et al., 2007; Welsch et al., 2007; Chukkapalli et al., 2008, 2010; Chu et al., 2010; Hamard-Peron et al., 2010; Chukkapalli and Ono, 2011). Biochemical, *in vivo*, *in vitro*, and genetic data have identified factors that modulate retroviral Gag-membrane interactions. Studies over the last decade have provided insights on the molecular and structural determinants of Gag-membrane binding. The human immunodeficiency virus type-1 (HIV-1) Gag polyprotein adopts a compact “folded over” conformation and exists in the monomeric or low-order oligomeric states prior to targeting to the PM (Datta et al., 2007, 2011; Kutluay and Bieniasz, 2010; Kutluay et al., 2014). Although, it is established that the nucleocapsid (NC) domain of Gag recognizes motifs in the viral RNA genome to mediate packaging, there is compelling evidence that the matrix (MA) domain also binds to cellular RNA to prevent premature Gag targeting to intracellular membranes (Chukkapalli et al., 2010, 2013; Chukkapalli and Ono, 2011; Hogue et al., 2012; Inlora et al., 2014; Kutluay et al., 2014; Olety and Ono, 2014). Upon transport of Gag to the PM, the interaction of MA with RNA is exchanged for an interaction of MA with PM lipids, inducing an extended conformation of Gag and formation of high-order Gag oligomers on the PM. The key to understanding this essential switch is elucidating at the molecular level the interaction of MA with specific PM components. Several retroviruses like Rous sarcoma virus (RSV), equine infectious anemia virus (EIAV), Mason-Pfizer monkey virus (M-PMV), and human T-lymphotropic virus type (HTLV-1) have evolved distinct mechanisms for Gag membrane targeting and assembly. Our understanding of retroviral Gag-PM interaction is incomplete because of the lack of molecular details on how membrane components contribute to the overall membrane binding. Eight out of the nine articles discuss the latest understanding of retroviral Gag-membrane binding. Prchal et al. review the latest developments on the characterization of Mason-Pfizer monkey virus (M-PMV) Gag interactions with the PM (Prchal et al., 2014). M-PMV, which belongs to *Betaretroviruses*, first assembles into virus-like particles (VLPs) in the pericentriolar region of the infected cell and therefore. Structural details of M-PMV MA binding to single phospholipids are discussed. Dick et al. describe the principles that govern Gag interactions with membranes, focusing on RSV and HIV-1 Gag (Dick and Vogt, 2014). The review defines lipid and membrane behavior, and discusses the complexities in determining how lipid and membrane behavior impact Gag membrane binding. Yandrapalli et al. review the role of plasma membrane lipids in HIV-1 Gag targeting and assembly, mainly focusing on membrane biophysics (Yandrapalli et al., 2014). Studies identified the 1,4,5-inositol trisphosphate receptor (IP_3_R), a channel mediating release of Ca^2+^ from ER stores, as a cellular factor differentially associated with HIV-1 Gag that might facilitate ESCRT function in virus budding. In a research article, Ehrlich et al. show that Gag modulates ER store gating and refilling (Ehrlich et al., 2014). It is shown that Gag accumulation at the plasma membrane required continuous IP_3_R activation. Elevation of Ca^2+^ level in the immediate vicinity of the plasma membrane is suggested to drive events that lead to stable membrane localization of assembling Gag. In their review, Alfadhli et al. focus on the functions of retroviral MA proteins, with an emphasis on the nucleic acid-binding capability of the HIV-1 MA protein and its effects on membrane binding (Alfadhli and Barklis, 2014). A review by Maldonado et al. discusses not only retroviral Gag-membrane interactions but also how Gag-Gag interactions contribute to the overall assembly process (Maldonado et al., 2014). Differences among retroviruses in Gag–Gag and Gag–membrane interactions implying various molecular aspects of the viral assembly pathway are described. Mariani et al. discuss the role of Gag and lipids during HIV-1 assembly in CD^4+^ T cells and macrophages (Mariani et al., 2014). Whereas, HIV-1 assembly and budding in macrophages is thought to follow the same general Gag-driven mechanism as in T-lymphocytes, the HIV-1 cycle in macrophage exhibits specific features. How Gag interacts with membrane lipids and what are the mechanisms involved in the interaction between the different membrane nanodomains within the assembly platform are not fully understood. Vlach et al. discuss the structural and molecular determinants of HIV-1 Gag binding to the plasma membrane (Vlach and Saad, 2015). This review emphasizes the structural findings on HIV-1 and HIV-2 MA binding to PM lipids and how these studies may advance our understanding of the overall Gag-membrane binding mechanism. The ninth article of this issue discusses the assembly and budding mechanisms of filoviruses including Marbug (MARV) and EBOV viruses (Stahelin, 2014). EBOV budding occurs from the inner leaflet of the plasma membrane (PM) and is driven by the matrix protein VP40, which binds to anionic lipid membranes. The review by Stahelin describes what is known regarding VP40 membrane interactions and what answers will fill the gaps. Collectively, the articles published under this special topic remarkably enriched our understanding of the role of membrane lipids during assembly, egress and release.

## Conflict of interest statement

The authors declare that the research was conducted in the absence of any commercial or financial relationships that could be construed as a potential conflict of interest.
